# Efficient Molecular Marker Design Using the MaizeGDB Mo17 SNPs and Indels Track

**DOI:** 10.1534/g3.114.010454

**Published:** 2014-04-17

**Authors:** A. Mark Settles, Alyssa M. Bagadion, Fang Bai, Junya Zhang, Brady Barron, Kristen Leach, Janaki S. Mudunkothge, Cassandra Hoffner, Saadia Bihmidine, Erin Finefield, Jaime Hibbard, Emily Dieter, I. Alex Malidelis, Jeffery L. Gustin, Vita Karoblyte, Chi-Wah Tseung, David M. Braun

**Affiliations:** * Horticultural Sciences Department and Plant Molecular and Cellular Biology Program, University of Florida, Gainesville, Florida 32611; † Division of Biological Sciences, Interdisciplinary Plant Group and the Missouri Maize Center, University of Missouri, Columbia, Missouri 65211; ‡ Biology Department, Saint Michael’s College, Colchester, Vermont 05439

**Keywords:** *Zea mays*, insertion–deletion polymorphism, genetic mapping, molecular marker, positional cloning

## Abstract

Positional cloning in maize (*Zea mays*) requires development of markers in the region of interest. We found that primers designed to amplify annotated insertion–deletion polymorphisms of seven base pairs or greater between B73 and Mo17 produce polymorphic markers at a 97% frequency with 49% of the products showing co-dominant fragment length polymorphisms. When the same polymorphisms are used to develop markers for B73 and W22 or Mo17 and W22 mapping populations, 22% and 31% of markers are co-dominant, respectively. There are 38,223 Indel polymorphisms that can be converted to markers providing high-density coverage throughout the maize genome. This strategy significantly increases the efficiency of marker development for fine-mapping in maize.

Maize whole genome sequencing has greatly simplified positional cloning of mutant loci and quantitative traits. Maize has very high levels of nucleotide sequence diversity (Chia *et al.* 2012; Jiao *et al.* 2012), which has been leveraged to develop multiple molecular marker platforms (Sharopova *et al.* 2002; Fu *et al.* 2006; Gore *et al.* 2009; Liu *et al.* 2010; Frascaroli *et al.* 2013; Qu and Liu 2013; Xu *et al.* 2013). These marker sets enable mapping of phenotypic loci, but the primary challenges to positional cloning are efficient identification of recombinant chromosomes and rapid development of molecular markers in the region of interest. A typical positional cloning project begins by using one of the developed and low-cost mapping platforms that rely on single nucleotide polymorphisms (SNPs) to map the locus (Jander *et al.* 2002; Liu *et al.* 2010). A large mapping population is then screened to identify recombinant individuals within the region. Finally, high-density markers are needed to narrow the area to a few candidate genes.

Flexible and scalable marker systems utilizing simple sequence repeat (SSR) or insertion–deletion (Indel) polymorphisms are a common choice for fine-mapping a single locus (Phillips *et al.* 2011; Slewinski *et al.* 2012). SSR tracks can be identified in the B73 genome sequence, but these require experimental testing to determine if individual markers will be useful for different mapping applications (Martin *et al.* 2010). Indel markers can be identified at low frequency by amplifying PCR products spanning introns or 3′ UTR of annotated genes (Fu *et al.* 2006).

The Mo17 inbred was sequenced by the Department of Energy Joint Genome Institute (JGI) with SNP and Indel polymorphisms annotated on the B73 genome (Sen *et al.* 2009). Markers designed from these annotations should be polymorphic for B73/Mo17 mapping populations and are expected to have a high frequency of polymorphism when either B73 or Mo17 is used as a mapping parent. Here, we show that PCR primers designed to amplify annotated B73/Mo17 Indel polymorphisms produce a high frequency of co-dominant and dominant molecular markers that can be scored using agarose gel electrophoresis. This marker development strategy enables rapid fine-mapping of mutants in any laboratory equipped for basic molecular biology techniques.

## Materials and Methods

**Figure 1:**
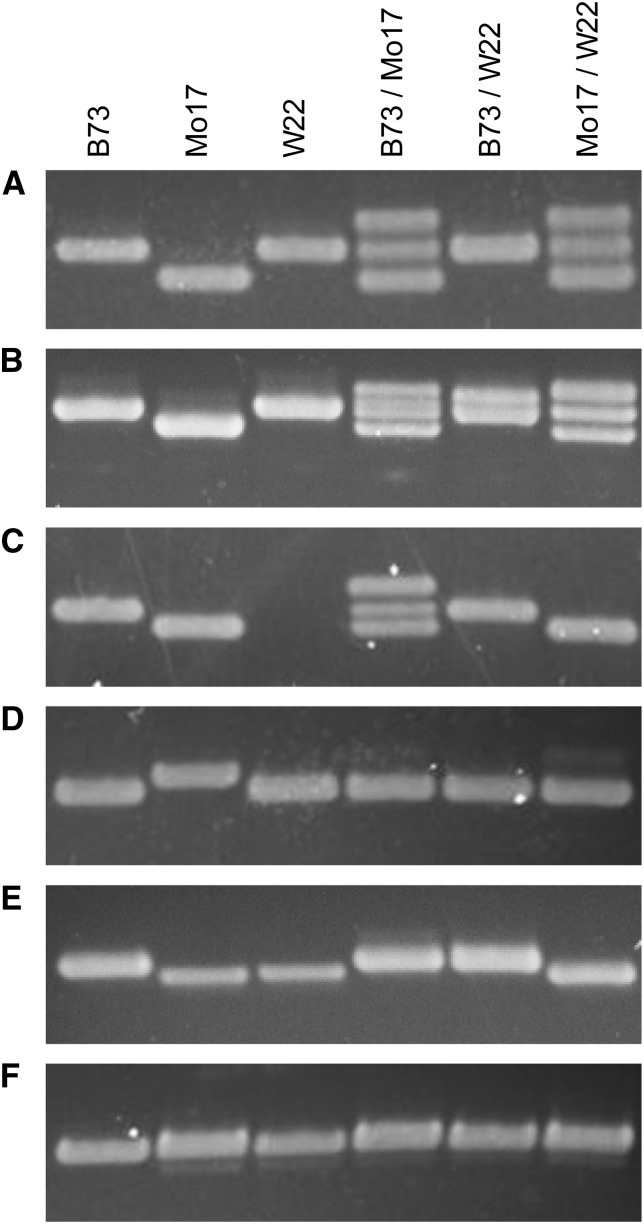
Examples of Indel markers designed from the Mo17 polymorphism track of MaizeGDB. (A) Marker ufIDP1-256.9 shows the most common co-dominant marker class, with B73 and W22 sharing the same size allele and each having a co-dominant polymorphism with Mo17. The B73 product was designed to amplify a 154-bp product, with Mo17 having a 17-bp deletion. (B) The ufID6-133.83 marker shows an Indel that amplified three different co-dominant alleles with a 255-bp B73 product and a 19-bp Mo17 deletion. (C) The ufIDP7-16.1 marker shows a co-dominant polymorphism between B73 and Mo17, whereas W22 shows PAV polymorphisms with both B73 and Mo17. The B73 product was designed to amplify a 143-bp product with a 13-bp Mo17 deletion. (D) The ufIDP1-257.3 marker amplifies a 168-bp product from B73 and W22 with a 14-bp insertion in Mo17. The B73 and W22 products are dominant over the Mo17 product. (E) The ufIDP4-20.905 marker amplifies a 201-bp product from B73 with an 11-bp deletion in Mo17 and W22. The B73 allele is dominant. (F) The ufIDP4-25.42 primers amplify a 192-bp product from all three inbred lines despite a 24-bp deletion predicted for Mo17 based on the polymorphism track.

MaizeGDB (www.maizegdb.org) provided the complete Mo17 polymorphism track based on the JGI sequence, which contains more than 4.5 million SNP and Indel polymorphisms. We selected the subset of 38,223 Indel polymorphisms with at least a 7-bp difference between the B73 and Mo17 alleles to design PCR primers for fine-mapping (Supporting Information, Table S1). Primer pairs were designed to amplify PCR products spanning 330 annotated Indels with expected B73 PCR products ranging from 80 to 300 bp in length. All primers selected were specific to the target B73 locus based on BLASTn searches of the maize genome assembly release 5b.60 and were tested using inbred DNA from B73 and Mo17. Subsets of primer pairs were tested for amplification of W22 inbred as well as F_1_ or mixed DNA from B73/Mo17, B73/W22, or Mo17/W22 to determine if the markers were co-dominant (Figure 1). PCR and 4% agarose gel electrophoresis were completed as described by Martin *et al.* (2010).

## Results and Discussion

We tested 330 markers for mapping 15 genetic loci to sample nearly 334 Mbp of the genome (Table S2). A B73 product amplified from all primer pairs tested, and a Mo17 allele amplified for 240 of the 330 markers. The PCR primer design strategy did not account for nearby Mo17 SNPs and short Indels. It is possible that Mo17 alleles failed to amplify in 90 loci due to nucleotide divergence between the B73 primer sequences and the Mo17 loci targeted. For the 240 markers where both B73 and Mo17 alleles amplified, we evaluated whether the expected size differences were observed based on the annotated insertion or deletion (Figure 1). Thirty markers amplified unexpected products from Mo17. In 12 cases, deletion polymorphisms amplified a larger Mo17 product or insertion polymorphisms amplified a smaller Mo17 product. For the remaining 18 markers, the Mo17 and B73 PCR products were the same size (Figure 1F). These data indicate a low level of disagreement (10% of Indel annotations) between the genome annotation and experimental evidence.

We tested 259 markers with B73/Mo17 DNA (Table 1). Of these, 49% amplified both B73 and Mo17 alleles as co-dominant fragment length polymorphisms (Figure 1, A–C). The small size differences between the B73 and Mo17 alleles resulted in frequent formation of slow migrating, heteroduplex bands in the B73/Mo17 PCR products. An additional 48% of the markers amplified only one allele in the B73/Mo17 DNA and were scored as dominant or presence–absence variants (PAV) (Figure 1, D and E). In all but one case, the B73 allele preferentially amplified over the Mo17 allele, which is consistent with the primers being designed from the B73 reference genome. Only 3% of markers were not polymorphic (Figure 1F).

**Table 1:** Summary of Indel markers tested using B73/Mo17, B73/W22, and Mo17/W22 DNA

Inbred Pair	No. Markers Tested	Co-Dominant	PAV	Dominant	Not Polymorphic	% Co-Dominant
B73/Mo17	259	127	80	44	8	49
B73/W22	201	44	36	16	105	22
Mo17/W22	201	63	48	20	70	31

Fu *et al.* (2006) reported a strategy to generate Indel markers between B73 and Mo17 by amplifying intron or 3′ UTR sequences. Slightly more than 7% of nearly 12,000 PCR products showed PAV polymorphism, and an additional 3.5% of the products showed distinct fragment length polymorphisms. This latter set of length polymorphisms was not tested for co-dominance in F_1_ or mixed DNA. A subset of these markers would be expected to show dominant amplification similar to that shown in Figure 1, D and E. Thus, designing markers from the Mo17 polymorphism track yields at least 14-fold higher frequency of co-dominant markers than the Fu *et al.* (2006) strategy.

Many public mutagenesis resources utilized the W22 inbred background for mutagenesis (Cowperthwaite *et al.* 2002; Till *et al.* 2004; Kolkman *et al.* 2005; McCarty *et al.* 2005; Ahern *et al.* 2009). To determine how readily B73/Mo17 Indel polymorphisms can be applied to fine-mapping with a W22 genetic background, we screened 201 markers for co-dominant polymorphisms in both the B73/W22 and Mo17/W22 inbred pairs (Table 1). The W22 allele was frequently the same length as the B73 allele with 52% of markers amplifying the same size product (Figure 1A), whereas 35% of the markers amplified the same size allele from both W22 and Mo17 DNA. Polymorphic markers for these two sets of inbred parents were divided equally between co-dominant and dominant/PAV polymorphisms (Table 1). Overall, 86% of the 201 Indel markers tested for B73/W22 and Mo17/W22 were polymorphic with at least one pair. Compared to SSR markers, only 43% of 4083 randomly selected SSR tracks were found to be polymorphic among 11 diverse inbred lines (Sharopova *et al.* 2002). More recently, next-generation sequencing of hundreds of inbred lines has identified thousands of SSRs with length polymorphisms predicted at a similar frequency to that observed by Sharopova *et al.* (2002) (Qu and Liu 2013; Xu *et al.* 2013). To identify SSR loci with as high a frequency of polymorphic markers as found in the B73/Mo17 Indel annotation, specific comparisons between mapping population parents would be needed.

Based on our review of current public resources, we believe Table S1 provides polymorphisms with the highest likelihood for successful development of new co-dominant amplified fragment length markers as long as either the B73 or the Mo17 inbred is a parent for the mapping population of interest. The average distance between potential markers in Table S1 is 53.7 kb, with a median distance of 10 kb between polymorphisms. Larger distances between polymorphisms coincide with centromere and heterochromatic regions of the chromosomes. Thus, polymorphism density is highest in gene-rich regions, and it is expected that the polymorphisms in Table S1 should provide sufficient coverage to fine-map any trait to a few candidate genes.

No trends were found for B73/Mo17 Indel marker co-dominant polymorphism rates based on the size of the Indel or the size of the expected B73 product. Heteroduplex products allow heterozygous recombinant individuals to be readily scored on agarose gels even if the Indel has a small size difference. However, a smaller (<150 bp) PCR product will make smaller size differences (<10 bp Indel) between contrasting homozygous alleles easier to resolve in agarose gels. Key design concerns for successful marker development are ensuring that the PCR primer sequences are specific for the target Indel locus and that the expected PCR product is within the range of resolution for a 4% agarose gel. To further improve the successful development of co-dominant markers, primer design could also take into account linked Mo17 SNP and short Indel polymorphisms. Our current fine-mapping strategy is to select five evenly spaced polymorphisms in the mapping interval to identify one to three co-dominant markers that refine the interval. We then repeat the process with evenly spaced polymorphisms within refined intervals until a suitably small region is identified for sequencing candidate genes.

## Supplementary Material

Supporting Information

## References

[bib1] Ahern K. R. Deewatthanawong P. Schares J. Muszynski M. Weeks R., 2009 Regional mutagenesis using Dissociation in maize. Methods 49: 248–254 19394430 10.1016/j.ymeth.2009.04.009

[bib2] Chia J. M. Song C. Bradbury P. J. Costich D. de Leon N., 2012 Maize HapMap2 identifies extant variation from a genome in flux. Nat. Genet. 44: 803–807 22660545 10.1038/ng.2313

[bib3] Cowperthwaite M. Park W. Xu Z. Yan X. Maurais S. C., 2002 Use of the transposon Ac as a gene-searching engine in the maize genome. Plant Cell 14: 713–726 11910016 10.1105/tpc.010468 PMC150591

[bib4] Frascaroli E. Schrag T. A. Melchinger A. E., 2013 Genetic diversity analysis of elite European maize (Zea mays L.) inbred lines using AFLP, SSR, and SNP markers reveals ascertainment bias for a subset of SNPs. Theor. Appl. Genet. 126: 133–141 22945268 10.1007/s00122-012-1968-6

[bib5] Fu Y. Wen T. J. Ronin Y. I. Chen H. D. Guo L., 2006 Genetic dissection of intermated recombinant inbred lines using a new genetic map of maize. Genetics 174: 1671–1683 16951074 10.1534/genetics.106.060376 PMC1667089

[bib6] Gore M. A. Chia J. M. Elshire R. J. Sun Q. Ersoz E. S., 2009 A first-generation haplotype map of maize. Science 326: 1115–1117 19965431 10.1126/science.1177837

[bib7] Jander G. Norris S. R. Rounsley S. D. Bush D. F. Levin I. M., 2002 Arabidopsis map-based cloning in the post-genome era. Plant Physiol. 129: 440–450 12068090 10.1104/pp.003533 PMC1540230

[bib8] Jiao Y. Zhao H. Ren L. Song W. Zeng B., 2012 Genome-wide genetic changes during modern breeding of maize. Nat. Genet. 44: 812–815 22660547 10.1038/ng.2312

[bib9] Kolkman J. M. Conrad L. J. Farmer P. R. Hardeman K. Ahern K. R., 2005 Distribution of Activator (Ac) throughout the maize genome for use in regional mutagenesis. Genetics 169: 981–995 15520264 10.1534/genetics.104.033738 PMC1449104

[bib10] Liu S. Chen H. D. Makarevitch I. Shirmer R. Emrich S. J., 2010 High-throughput genetic mapping of mutants via quantitative SNP-typing. Genetics 184: 19–26 19884313 10.1534/genetics.109.107557 PMC2815916

[bib11] Martin F. Dailey S. Settles A. M., 2010 Distributed simple sequence repeat markers for efficient mapping from maize public mutagenesis populations. Theor. Appl. Genet. 121: 697–704 20401644 10.1007/s00122-010-1341-6

[bib12] McCarty D. R. Settles A. M. Suzuki M. Tan B. C. Latshaw S., 2005 Steady-state transposon mutagenesis in inbred maize. Plant J. 44: 52–61 16167895 10.1111/j.1365-313X.2005.02509.x

[bib13] Phillips K. A. Skirpan A. L. Liu X. Christensen A. Slewinski T. L., 2011 *vanishing tassel2* encodes a grass-specific tryptophan aminotransferase required for vegetative and reproductive development in maize. Plant Cell 23: 550–566 21335375 10.1105/tpc.110.075267 PMC3077783

[bib14] Qu J. Liu J., 2013 A genome-wide analysis of simple sequence repeats in maize and the development of polymorphism markers from next-generation sequence data. BMC Res. Notes 6: 403 24099602 10.1186/1756-0500-6-403 PMC3828028

[bib15] Sen T. Z. Andorf C. M. Schaeffer M. L. Harper L. C. Sparks M. E., 2009 MaizeGDB becomes ’sequence-centric’. Database 2009: bap020 21847242 10.1093/database/bap020 PMC2964019

[bib16] Sharopova N. McMullen M. D. Schultz L. Schroeder S. Sanchez-Villeda H., 2002 Development and mapping of SSR markers for maize. Plant Mol. Biol. 48: 463–481 12004892 10.1023/a:1014868625533

[bib17] Slewinski T. L. Baker R. F. Stubert A. Braun D. M., 2012 *Tie-dyed2* encodes a callose synthase that functions in vein development and affects symplastic trafficking within the phloem of maize leaves. Plant Physiol. 160: 1540–1550 22932757 10.1104/pp.112.202473 PMC3490577

[bib18] Till B. J. Reynolds S. H. Weil C. Springer N. Burtner C., 2004 Discovery of induced point mutations in maize genes by TILLING. BMC Plant Biol. 4: 12 15282033 10.1186/1471-2229-4-12 PMC512284

[bib19] Xu J. Liu L. Xu Y. Chen C. Rong T., 2013 Development and characterization of simple sequence repeat markers providing genome-wide coverage and high resolution in maize. DNA Res. 20: 497–509 23804557 10.1093/dnares/dst026 PMC3789560

