# Supplementary material for: Efficient Molecular Marker Design Using the MaizeGDB Mo17 SNPs and Indels Track
Source: G3 (Bethesda). 2014 Apr 17;4(6):1143–5. doi: 10.1534/g3.114.010454 (PMC4065257; doi:10.1534/g3.114.010454)
Supplement: Supporting Information [file supp_4_6_1143__index.html]

Efficient Molecular Marker Design Using the MaizeGDB Mo17 SNPs and Indels Track — Supporting Information 

# Efficient Molecular Marker Design Using the MaizeGDB Mo17 SNPs and Indels Track

## Supporting Information for Settles *et al.*, 2014

**Files in this Data Supplement:**

- Table S1 - Insertion-Deletion (Indel) polymorphisms of 7 bp or greater from the MaizeGDB Mo17 polymorphism track. (.zip, 2 MB)
- Table S2 - Indel markers tested in this study. (.zip, 70 KB)
